# Comparative analysis of the daily brain transcriptomes of Asian particolored bat

**DOI:** 10.1038/s41598-022-07787-z

**Published:** 2022-03-09

**Authors:** Guoting Zhang, Yujia Chu, Tinglei Jiang, Jingjing Li, Lei Feng, Hui Wu, Hui Wang, Jiang Feng

**Affiliations:** 1 College of Life Science, Jilin Agricultural University, Changchun, 130118 China; 2 Jilin Provincial Key Laboratory of Animal Resource Conservation and Utilization, Northeast Normal University, Changchun, 130117 China

**Keywords:** Molecular biology, Zoology

## Abstract

Daily rhythms are found in almost all organisms, and they comprise one of the most basic characteristics of living things. Daily rhythms are generated and mainly regulated by circadian clock. Bats have attracted interest from researchers because of their unique biological characteristics. However, little is known about the molecular underpinnings of daily rhythms in bats. In this study, we used RNA-Seq to uncover the daily rhythms of gene expression in the brains of Asian particolored bats over the 24-h day. Accordingly, four collected time points corresponding to four biological states, rest, sleep, wakefulness, and active, were selected. Several groups of genes with different expression levels in these four states were obtained suggested that different physiological processes were active at various biological states, including drug metabolism, signaling pathways, and the circadian rhythm. Furthermore, downstream analysis of all differentially expressed genes in these four states suggested that groups of genes showed daily rhythms in the bat brain. Especially for *Per1*, an important circadian clock gene was identified with rhythmic expression in the brain of Asian particolored bat. In summary, our study provides an overview of the brain transcriptomic differences in different physiological states over a 24-h cycle.

## Introduction

Daily rhythm is an important circadian rhythm and is a distinct feature of a wide range of organisms, especially for mammals^1,2^. Various biological processes including body temperature cycling, hormone release, heartbeat, blood pressure changes, and sleep–wake cycles, etc., exhibit daily rhythms^1,3,4^. It is generally assumed that daily rhythms enable organisms to adapt to the daily fluctuations in environmental factors driven by the rotation of the earth around its axis, these rhythms are driven by circadian clocks^2,5,6^. The circadian clock in mammals is based on an interlocked feedback loop that regulates the expression patterns of major circadian rhythm-related genes, including core clock genes (*Per1/2, Cry1/2*) and clock output genes^6,7^.

Essentially all organisms have evolved daily rhythms in physiology, behavior and gene expression to adapt to a dynamic environment^3^. In mammals, endogenous physiological and behavioral processes are intimately linked with the function of circadian clocks and oscillators distributed throughout the brain and body^8^. The core circadian clock genes are expressed in the brain and some of them are showed daily expression rhythms. In addition, circadian clock genes could also regulate daily rhythms expression of coordinated genes in the brain^6^. Daily cycles in behavior, physiology, and metabolism are presumed to arise mainly from coordinated gene expression rhythms in brain regions^8,9^. These physiological changes coincide with oscillating gene expression patterns that are coordinated to adapt cellular activity to periods of activity and inactivity^2^. In addition to the circadian clock genes, other environmental signals, such as light periods, feeding patterns, and environment temperature, could also influence the daily gene expression patterns in the brain and other tissues.

Studies on daily rhythms of biological processes, behavior and also gene expression generally focus on model organisms such as mice, while little is known about other wild nocturnal animals. As totally wild nocturnal mammals, bats are more enigmatic and are known for their abilities to fly and echolocate in the dark^10^. Bats belong to the order Chiroptera, the second largest group of mammals after Rodentia, and they have different diurnal activities^11^. Despite numerous studies concerning bats’ daily behavior patterns and physiological processes, little is known about the daily rhythms of gene expression in their brain, which may provide insights into the genetic basis for the daily rhythms of biological processes and behavior.

To gain insights into the daily rhythms in gene expression of bat brain, we selected the Asian particolored bat (*Vespertilio sinensis*) as the research object. Asian particolored bats belong to the family *Vespertilionidae* and are one of the most common bat species in northeast China^12^. They often dwell in the rooves or eaves of bridge holes or old buildings and are typical nocturnal echolocating bats. In this study, the comparative RNA-seq method was used to examine daily rhythms in brain gene expression in *V. sinensis* over a 24-h period. Four sampled time points corresponding to the four representative biological states, rest, sleep, wakefulness, and active, were selected. We aimed to 1) identify differentially expressed genes among the four different biological states; 2) determine the rhythmic changes of associated important physiological processes reveled by differentially expressed genes; and 3) identify potential circadian clock genes exhibiting cycling gene expression in 24-h cycle. Therefore, this study provides evidence for the daily rhythm in gene expression affecting the coordination of physiology and behavior in the brain of bat over a 24-h period.

## Results

### Sequence analysis, assembly, and functional annotation

The transcriptome sequencing results of brain tissue are shown in Table 1. After filtering the raw reads, 143,936,974, 142,787,930, 139,867,950 and 138,606,658 clean reads were obtained for the four brain states, respectively, and the corresponding base numbers were 22,640,739,600 bp, 22,425,822,300 bp, 22,051,944,000 bp and 21,898,530,000 bp (Table 1). The proportions of clean/raw reads of the four states were between 94.49% and 95.51%, suggesting the high quality of the RNA-Seq data available for further analyses.

**Table 1:** Sequencing and assembly statistics of brain samples for *V. sinensis.*

	Rest	Sleep	Wake	Activity
**Sequencing**				
Total reads (raw reads)	150,938,264	149,505,482	147,012,960	145,990,200
Total sequences (bp)	22,640,739,600	22,425,822,300	22,051,944,000	21,898,530,000
Clean reads	143,936,974	142,787,930	139,867,950	138,606,658
Ratio of clean/raw	95.36%	95.51%	95.13%	94.94%
**Assembly**				
Unigenes	403,707			
N50	615			
N90	244			
Max. length	19,402			
Min. length	201			
Ave. length	511			

The clean reads of this brain area and liver were assembled together, and a total of 403,707 unigenes sequences were obtained. The unigenes sequences were used as the reference genome in the subsequent analysis of the brain. The lengths of 403,707 unigenes ranged from 201 to 19,402 bp, with an average length of 511 bp, an N50 value of 615 bp, and an N90 value of 244 bp.

Among the 403,707 unigenes, 83,237, 60,588, 23,820 and 9,176 were respectively annotated by the NR, Swiss-Prot, KOG, and KEGG databases (Supplementary Fig. S1). For KOG annotation in particular, the term of signal transduction mechanisms was the most highly represented (Supplementary Fig. S2).

### DEGs analysis

The correlation analysis and principal component analysis (PCA) were conducted based on the TPM values. From the correlation results (Supplementary Fig. S3), the sample had good repeatability and can be used for gene differential expression analysis. The results of principal component analysis (PCA) showed four groups corresponding to the four time points (see Supplementary Fig. S4) that were indicative of the sampling having sufficient reproducibility and rationality.

**Figure 1:**
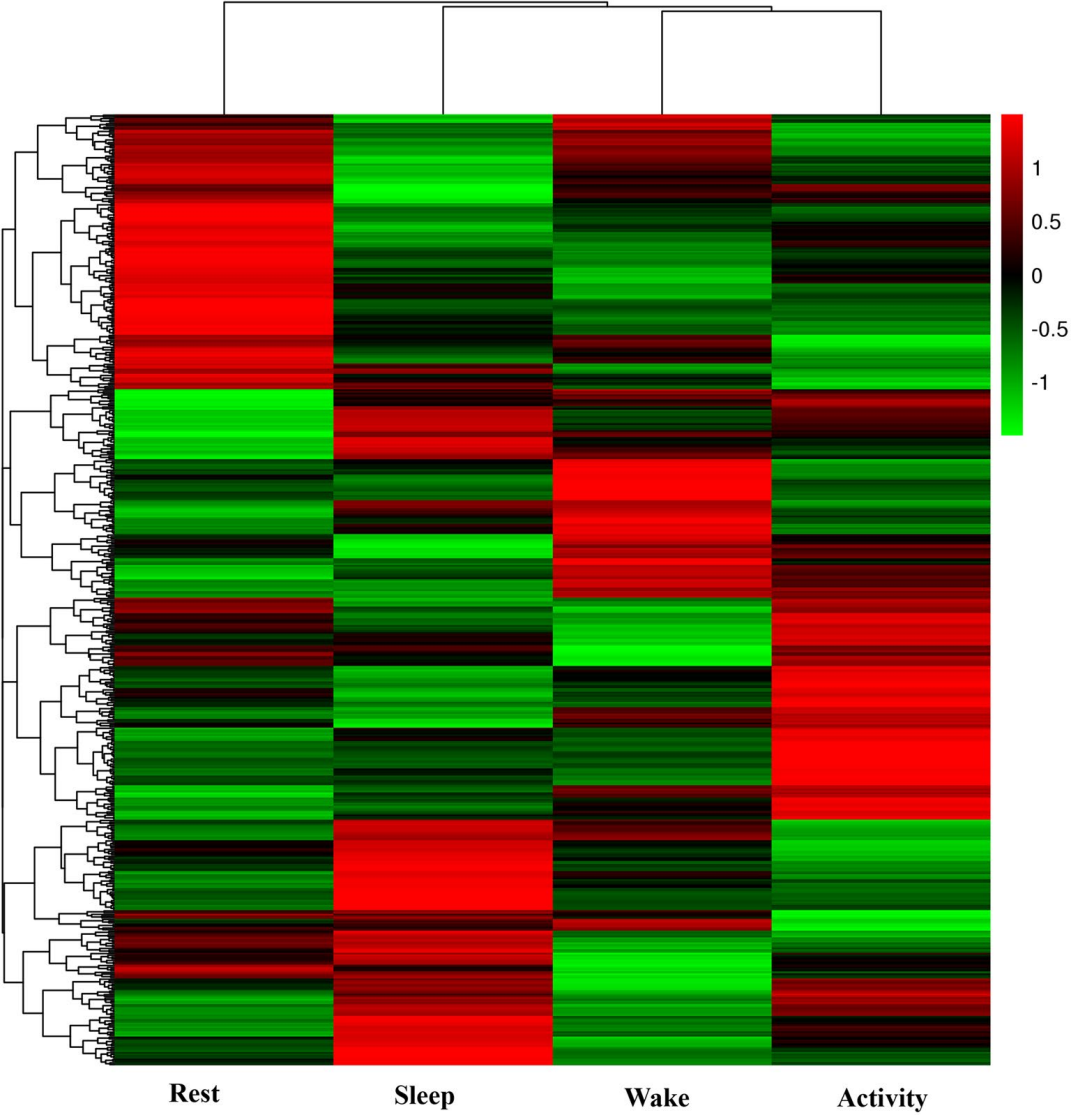
Heatmaps based on differentially expressed genes from six pairwise comparisons of four time points. Different colors indicate relative expression levels.

In the six pairwise comparisons, a total of 672 DEGs were detected (Table 2). In the rest vs activity comparison, the number of DEGs was the highest, with 136 genes, and the lowest number of DEGs was detected in the wake vs active comparison, with 60 genes. The numbers of upregulated genes in the comparison groups were higher than the numbers of downregulated genes in the two comparisons. In the wake vs active comparison, this may be because bats are always active after wakening to prepare for subsequent hunting behavior followed by a highly active predation state, so the numbers of DEGs in the two states were less, as similar genes were expressed. A heatmap of the hierarchical clustering of all DEGs indicated the large differences in gene expression between different time points (Fig. 1).

**Table 2:** The number of DEGs in the six pairwise comparisons of brain tissue.

Comparable group	Up	Down	Total
Rest vs sleep	63	59	122
Sleep vs wake	65	69	134
Wake vs activity	32	28	60
Rest vs activity	63	73	136
Rest vs wake	63	57	120
Sleep vs activity	47	53	100

### GO and KEGG analysis of DEGs

**Figure 2:**
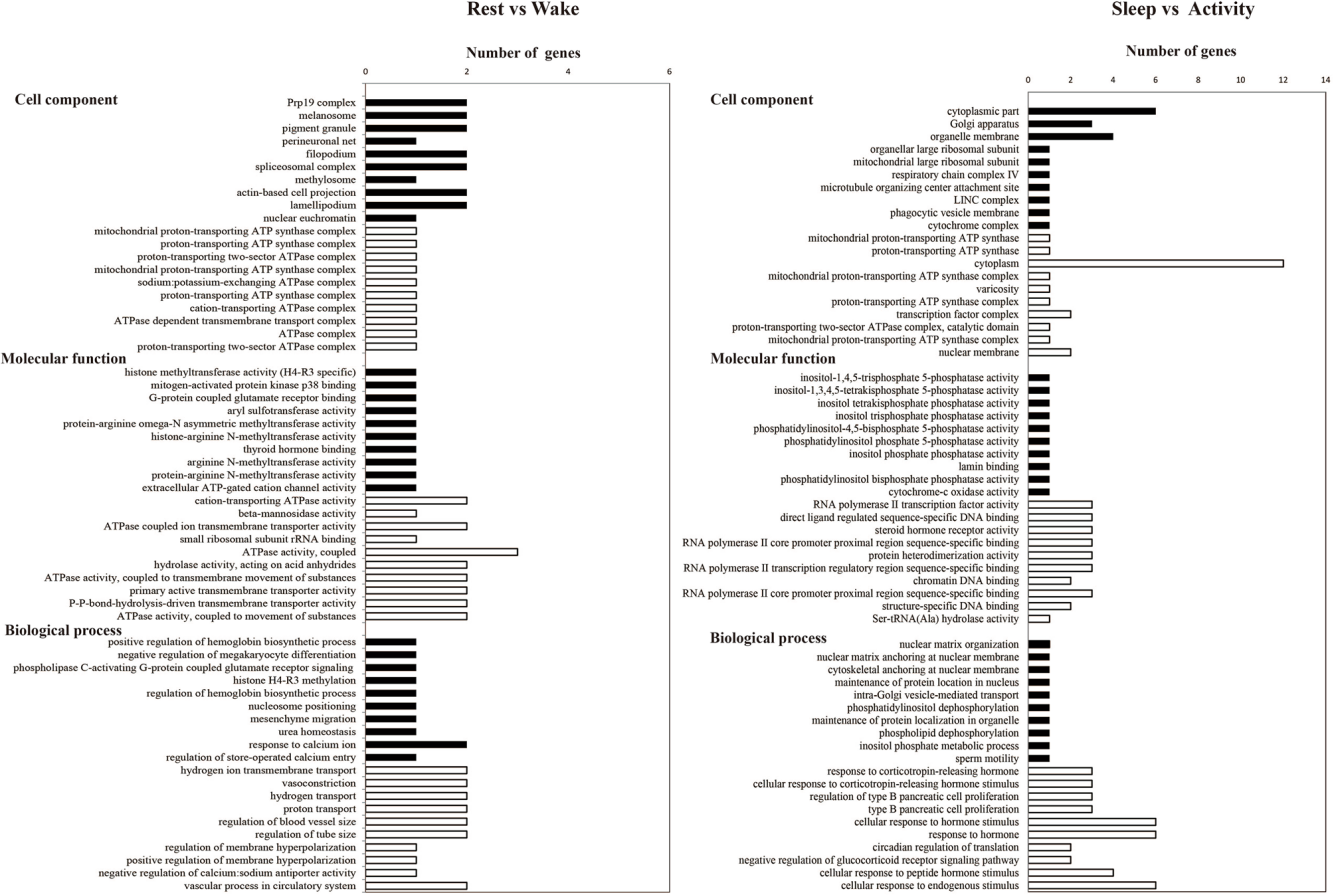
The top 10 GO categories with the most significant *p* values (cell component, molecular function, and biological process) were significantly enriched for upregulated and downregulated genes in rest vs wake and sleep vs activity comparisons. The black and white bars represent the terms significantly enriched for upregulated and downregulated genes, respectively.

**Figure 3:**
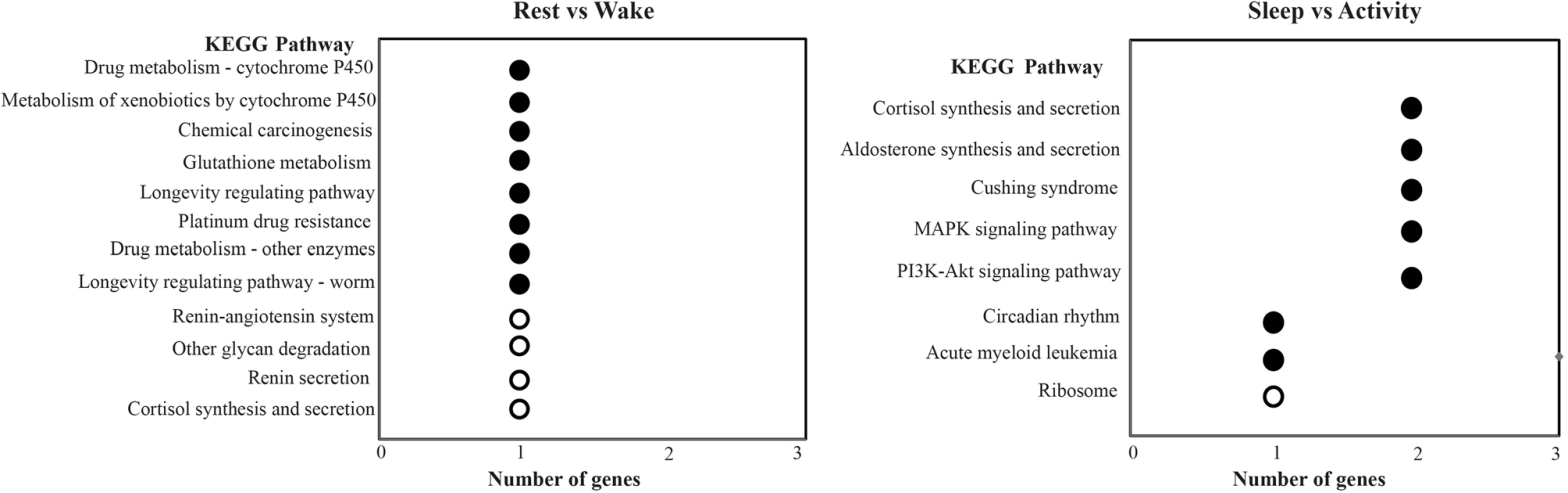
KEGG pathways significantly enriched for DEGs from rest vs wake and sleep vs activity comparisons. The black and white bubbles represent pathways that are significantly enriched for upregulated and downregulated genes, respectively.

The six pairwise comparisons were analyzed by GO and KEGG enrichment, and the results suggested that the bats had significant differences in the metabolic and transport activities at different time points during the day (Supplementary Tables S1–S4). The rest vs wake comparison represented the two extreme states before and after predation. There were 63 genes that showed high expression levels in the rest state and low expression levels in the wake state (Table 2). In the cell component of GO, multiple complexes were significantly enriched by these 63 genes, including Prp19 complex, spliceosomal complex, and DNA packaging complex. In the category of molecular function, these genes were significantly enriched in terms of transferase activity, for example, histone methyltransferase activity (H4-R3 specificity), aryl sulfotransferase activity, and protein-arginine omega-N asymmetric methyltransferase activity. In the category of biological process, these genes were significantly enriched in hemoglobin synthesis-related terms such as positive regulation of hemoglobin biosynthetic process, regulation of hemoglobin biosynthetic process, and hemoglobin biosynthetic process (Fig. 2). Regarding the KEGG results, various metabolic pathways were significantly enriched by these 63 genes, including drug metabolism-cytochrome P450, metabolism of xenobiotics by cytochrome P450, and glutathione metabolism (Fig. 3). The above results indicated that hemoglobin synthesis, transferase activity, and metabolic activity of *V. sinensis* in the rest state may be higher than during the wake state.

There were 57 genes that showed low expression levels in the rest state and high expression levels in the wake state. In the cell component category, these genes were significantly enriched in the terms of proton transport. The three top GO terms with the most significant *p* values were mitochondrial proton-transporting ATP synthase complex, proton-transporting ATP synthase complex, and proton-transporting two-sector ATPase complex, catalytic domain. In the molecular function category, these genes were significantly enriched in terms that were closely related to various enzyme transport pathways such as cation transporting ATPase activity, beta mannosidase activity, and ATPase coupled ion transmembrane transporter activity. For the biological process category, transport-related terms were significantly enriched by these 57 genes, including hydrogen ion transmembrane transport, hydrogen transport, and proton transport (Fig. 2). Regarding the KEGG results, a variety of endocrine-related pathways were significantly enriched by these 57 genes, including the renin-angiotensin system, renin secretion, and cortisol synthesis and secretion (Fig. 3). The above results indicated that the intensity of transport and endocrine activities of bats in the wake state may be higher than in the rest state.

In addition, sleep and activity were the two states that best reflected the difference between the day and night activities of *V. sinensis*. There were 47 genes that showed high expression levels in the sleep state and low expression levels in the active state (Table 2). In the cell component category, these genes were significantly enriched in organelle-related terms such as cytoplasmic part, Golgi apparatus, and organelle membrane. In the molecular function category, these genes were significantly enriched in terms related to enzyme activities, including inositol tetrakisphosphate phosphatase activity, cytochrome c oxidase activity, and heme-copper terminal oxidase activity. In the biological process category, these 47 genes were significantly enriched in nuclear-related terms including nuclear matrix organization, nuclear matrix anchoring at nuclear membrane, and cytoskeletal anchoring at nuclear membrane (Fig. 2). For the KEGG results, the 47 genes in the sleep state were only significantly enriched in the ribosome pathway (Fig. 3).

In addition, there were 53 genes that were expressed at low levels in the sleep state and highly expressed in the activity state. In the cell component category, terms that were related to proton transport were significantly enriched by the 53 genes, including mitochondrial proton-transporting ATP synthase, catalytic core, and proton transporting ATP synthase. In the molecular function category, genes were significantly enriched in the terms related to DNA binding, including RNA polymerase II transcription factor activity (ligand-activated sequence-specific DNA binding), transcription factor activity (direct ligand regulated sequence-specific DNA binding), and chromatin DNA binding. In the biological process category, these genes were significantly enriched in multiple terms related to endocrine activities, and the three top GO terms with the most significant *p* values were response to corticotropin releasing hormone, cellular response to corticotropin releasing hormone stimulus, and regulation of type B pancreatic cell proliferation. In addition, these genes were also significantly enriched in terms related to the circadian clock, including entrainment of circadian clock, circadian regulation of gene expression, and circadian rhythm (Fig. 2). In addition, in the biological process category of rest vs sleep and sleep vs wake comparisons, we also found that various terms related to light response were significantly enriched, including phototransduction, detection of visible light, and response to light stimulus (Supplementary Table S3).

Regarding the KEGG results for the 53 genes in the sleep vs activity comparison, various hormone secretion pathways and signal transduction pathways were significantly enriched by the 53 genes, including cortisol synthesis and secretion, MAPK signaling pathway, and PI3K-Akt signaling pathway. In addition, one circadian clock-related pathway, circadian rhythm was significantly enriched by the *Per1* gene (Fig. 3). The above results showed that the two states of bats do involve daily rhythm genes and regulate the regular changes of some circadian physiological processes.

### Downstream rhythmic analysis of DEGs

**Figure 4:**
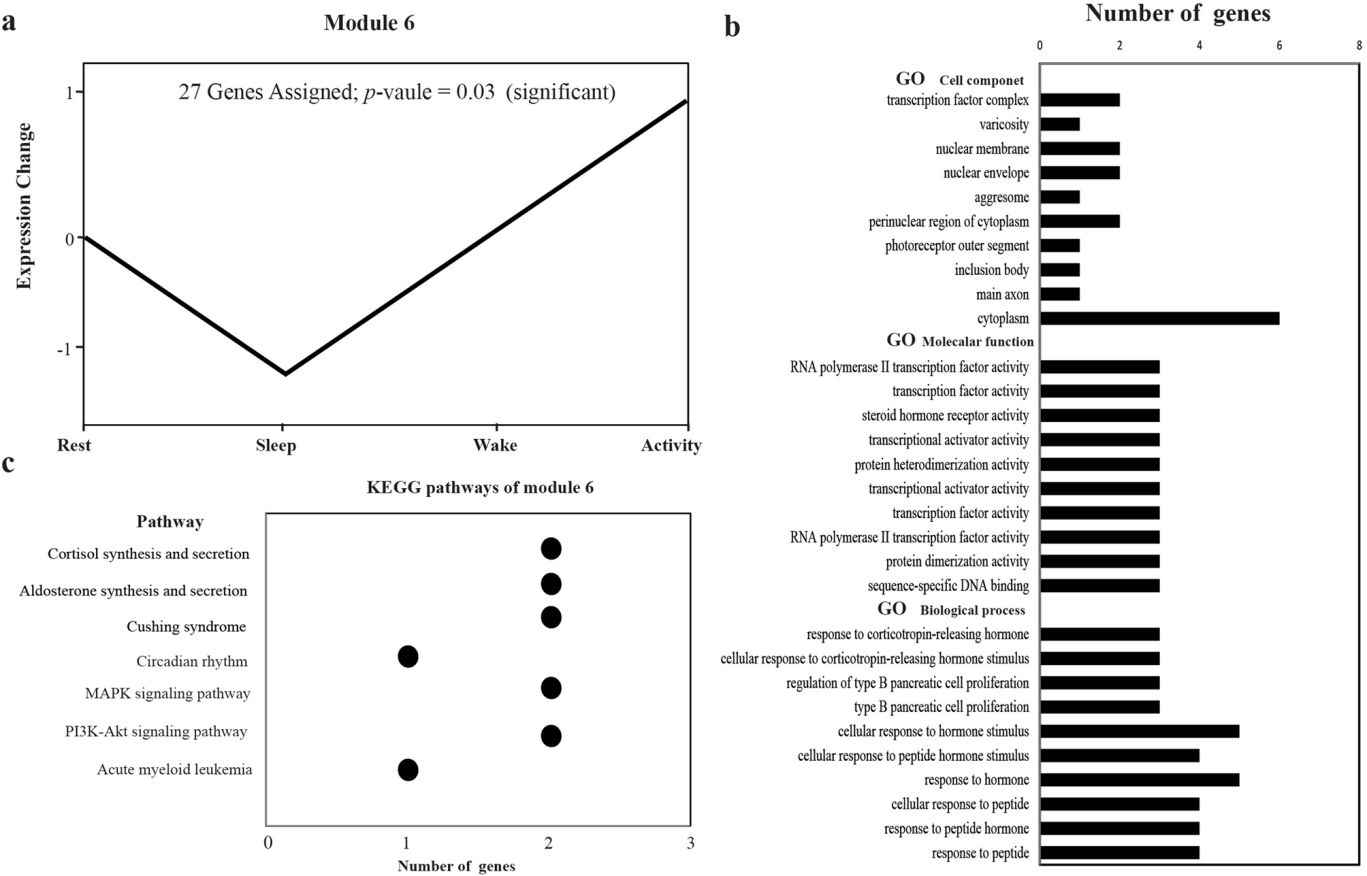
The trend of module 6 and the associated enrichment results. (**a**) The trend of genes in module 6 at four time points. (**b**)Top 10 GO categories with the most significant *p* values that were significantly enriched for genes from module 6. (**c**) KEGG pathways significantly enriched for genes from module 6.

To clarify the periodic expression and daily rhythmicity of gene expression in the brain area of *V. sinensis* during a 24-h period, we examined the union set of the six groups of DEGs, and a total of 557 genes were obtained. Then, we conducted trend analysis on these 557 genes, and the results are shown in Supplementary Fig. S5. Among the 20 modules obtained, the DEGs were significantly clustered in module 6 that contained 27 genes. These 27 genes had similar expression trends at the four time points during the day. The expression levels of these genes were decreased in the resting state, were the lowest in the sleep state, and gradually increased in the waking state, reaching the highest expression level in the activity state (Fig. 4a). A potential important circadian clock gene, *Per1* was found in module 6. These genes in module 6 were used in enrichment analysis, and the results are shown in Fig. 4 and Supplementary Table S5. Numerous pathways and terms were significantly enriched by these genes, including endocrine hormones, signal pathways, and circadian clock-related terms and pathways, etc. These genes showed obvious daily rhythms and were closely associated with bat activity states. Through the above analysis, we believe that the trend in gene expression changes for module 6 was consistent with the biological habits of bats. Bats in an active state require high expression levels of these genes, so the physiological and biochemical processes that these genes participate in play important roles in the activity and periodic awakening of bats.

In addition, there were six rhythmic genes were detected by ARSER analysis using the R metacycle package, however, no rhythmic genes were found by JTK_CYCLE analysis. Rhythmic gene expression patterns and the *p* values of the six genes were shown in Fig. 6. Among them, *LOC106694382*, *SYP*, *MAP1B* and *TTC1* genes were also grouped in module 6, and their expression patterns over the four time points of a day were similar to those genes showed in module 6 (Fig. S5 and Fig. 6).

### Important circadian clock genes

Combining the results of DEGs and trend analysis, we found that the genes in the sleep vs activity comparison and the genes in module 6 were enriched in pathways related to the circadian clock (Supplementary Tables S3 and S5). We further analyzed the genes in the pathways related to the circadian clock and found that they all involved *Per1*. *Per1* as an important circadian clock genes showed daily rhythms expression patterns in the bat brain, lower expression values during the sleep phase and higher expression values during the active phase. In the GO results of the sleep vs activity comparison and trend module 6, *Per1* was significantly enriched in terms related to the circadian clock. We marked the *Per1* gene in the figure illustrating the circadian rhythm pathway to show its important role in the pathway (Supplementary Fig. S6). Besides *Per1*, other circadian clock genes were also found in our transcriptome data, such as *Bmal1*, *Cyr1*, *Cyr2*, *Clock*, *Per2*, *Per3*, *Timeless*, however, with no significantly rhythmic expressed patterns over the four sampling time points over a day.

### Quantitative real-time PCR (qPCR) validation.

**Figure 5:**
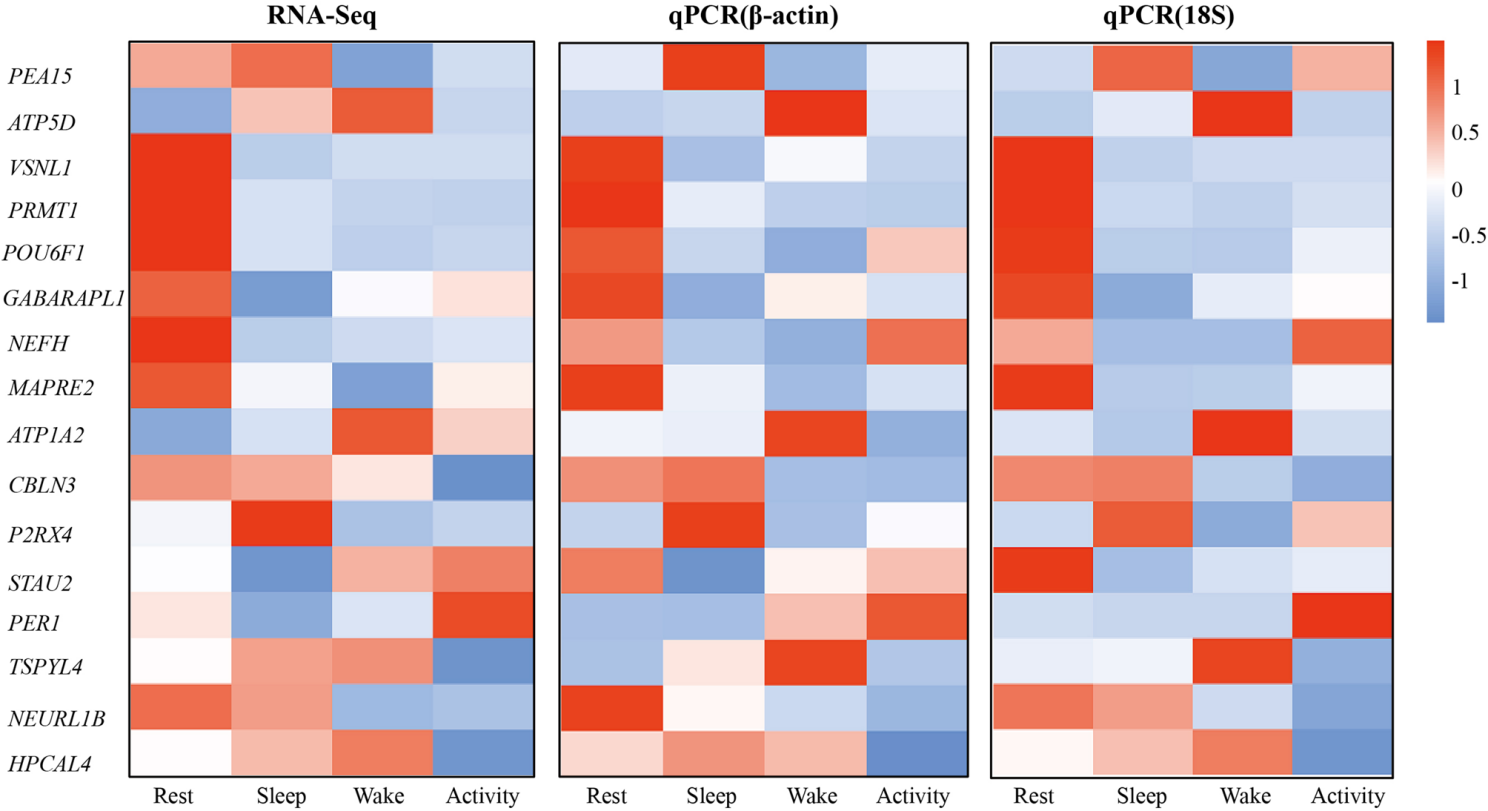
Comparisons of 16 differentially expressed genes by qPCR for technical validation. Heatmap from left to right representing log_2_ fold change expression values from RNA-Seq (TPM) and qPCR (using β-actin and 18S for normalisation). The correlation coefficient between log_2_ fold change expression values detected by RNA-Seq and qPCR was 0.455 (*p* < 0.01).

We compared the expression patterns of the 16 randomly selected genes detected by RNA-Seq with the results of qPCR experiments to test the validity of our measurements. The results conducted by RNA-Seq and qPCR experiments showed similar expression patterns (Fig. 5) with a significant correlation (*p* < 0.01), which verified the reliability of our RNA-Seq analysis.

## Discussion

**Figure 6:**
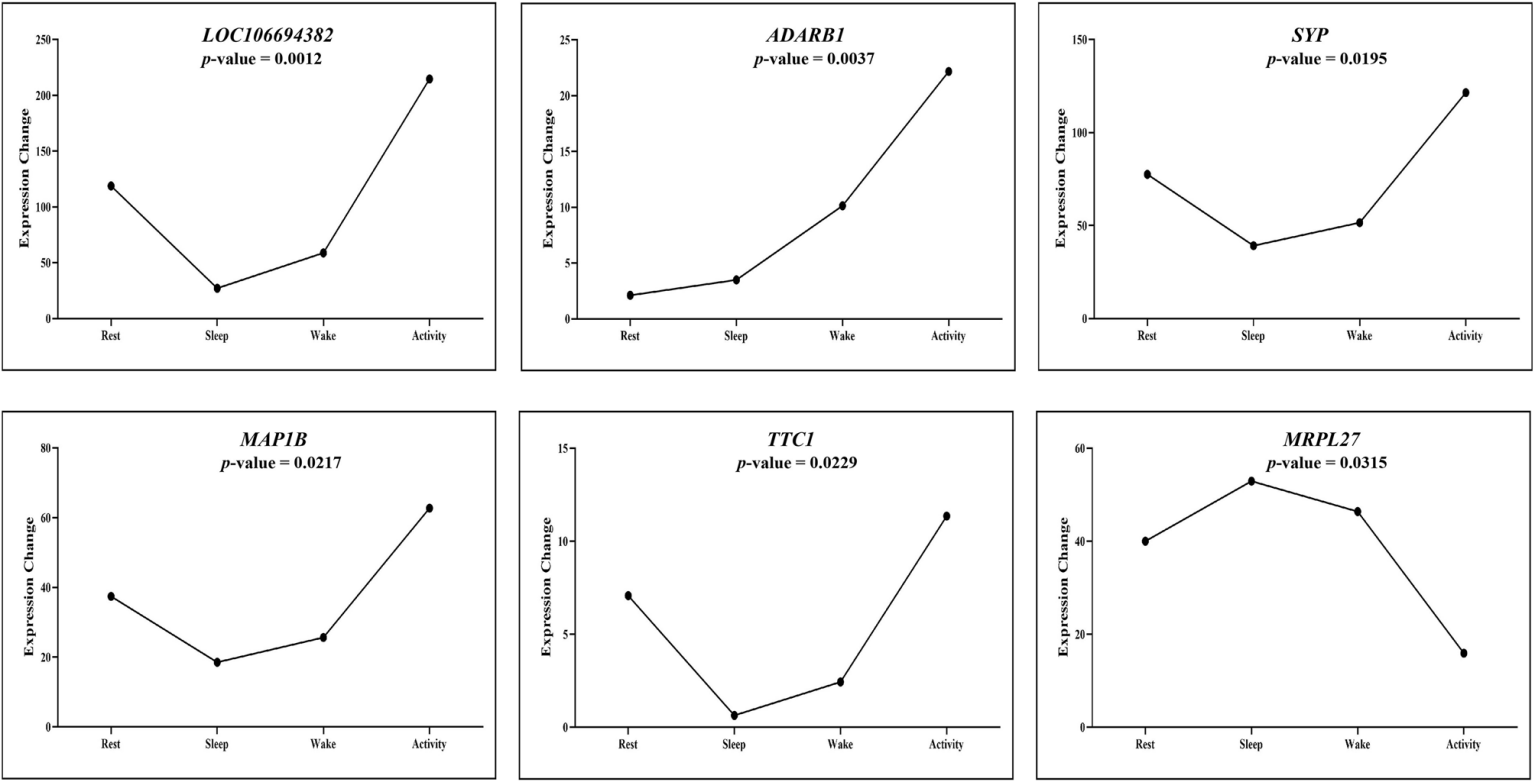
Expression patterns of the six rhythmic genes over the four time points of a day.

The daily rhythm is the result of animals adapting to the environment, and the daily variation of the physiological activities of animals is closely associated with the rhythmic expression of important genes. In this study, we used transcriptome sequencing technology to analyze the differences in genes expression of 4 biological states in the brains of *V. sinensis*, with a view to revealing the daily rhythm changes in gene expression in the brain of a nocturnal mammal. In addition, to clarify the differences in gene expression and physiological processes in the brain area of bats during sleep and activity, rest and wake, we focused on the sleep vs activity and rest vs wake comparisons and found that bats have different degrees of changes in corresponding physiological processes in different states. The analysis can not only reflect the daily changes in the physiological and biochemical activities of genes but also screen out genes that may be involved in regulating the core clock genes in the brain, thereby providing a reference for further understanding the mutual adaptations of animals and the environment, and at the same time enriching the research on the daily rhythms of nocturnal animals (Fig. 6).

### Circadian rhythms related physiological processes between sleep and activity states

Differentially expressed genes indicated significant differences in the physiological processes of bats in sleep vs activity. In the active state of *V. sinensis*, the pathways related to protein kinase signals are likely to be important circadian clock regulation pathways in the bat brain. The MAPK signaling pathway and the PI3K-Akt signaling pathway were significantly enriched by the genes with high expression levels in the active state. Previous studies have shown that in the mammalian SCN of the hypothalamus, the MAPK pathway can be used as an input pathway to allow the endogenous clock to enter the 24-h rhythm cycle^13^. The MAPK pathway can also interact with the components of the molecular circadian oscillator to affect the cycle of the circadian clock^13^. The PI3K-Akt signaling pathway can participate in the output pathway of animal retinal circadian rhythms^14^. The structure of the mammalian circadian clock system is composed of the input pathway of external signals, the oscillating central biological clock, and the output pathway of the rhythm^6^. Combining the results of this study, we speculate that these two signal pathways may be involved in the input and output pathways of the circadian clock system, and they play an important role in daily rhythm regulation mechanism in the brain of *V. sinensis*. In addition, genes with high expression levels in the active state were also involved in multiple pathways or terms related to the circadian clock such as circadian rhythm, circadian rhythm–fly, and circadian regulation of gene expression, indicating that the bat brain is in the optimal state for overall physiological process and the regulation of the circadian clock during the active state, and the daily rhythms of gene expression are likely regulated by the circadian clock.

When bats are active, the physiological processes related to various endocrine hormones in the brain are relatively active, for example cortisol synthesis and secretion and aldosterone synthesis and secretion. Cortisol is a glucocorticoid with an endogenous circadian rhythm driven by the central circadian pacemaker (CCP) of the SCN^15^. Studies have shown that cortisol levels in giant pandas show obvious circadian rhythm changes that drop to the lowest level in the middle of the night and reach a peak at dawn^16^. Cortisol has a wakeful response and the response refers to the increase in cortisol concentrations that occur within the first hour after awakening^15^. A superimposed pulsatile pattern of cortisol can allow the animal to have rapid responses that fine tune the body’s reaction to changes in the environment^17^. We speculate that bats also have a cortisol awakening response when they are awake and may maintain a higher concentration when they are in an active state and better adapt to the external environment. Aldosterone is a mineralocorticoid that plays an important role in regulating systemic blood pressure^18^. In rats, the concentration of aldosterone showed a clear circadian rhythm, and the concentration reached its peak at night and was the lowest at noon^19^. Studies have shown that the mineralocorticoid hormone aldosterone also plays an important role in avian osmoregulation^20^. When sunbirds (*Nectarinia talatala*) were in negative water balance, aldosterone concentrations and outputs at night were significantly higher than diurnal levels^20^. We speculate that the rhythm in *V. sinensis* may also resemble that of rodents and birds, with a higher concentration of aldosterone at night. Therefore, we suggest that the secretion of many hormones in bats occurs in the active state and prepares the bat for subsequent predation activity.

The results of KEGG and GO of 47 genes with high expression levels in the sleep state indicated that the physiological processes related to protein synthesis are relatively active in the brain of *V. sinensis*. In the KEGG results, the ribosome pathway was significantly enriched, and in biological process category of GO, terms such as mitochondrial ribosome, mitochondrial large ribosomal subunit, and organellar large ribosomal subunit were also significantly enriched. Previous studies indicated that the circadian oscillator regulates the transcription of ribosomal protein mRNA and ribosomal RNA^21^. The circadian clock can affect the time translation of some mRNAs involved in ribosome biogenesis by controlling the transcription of translation initiation factors and the clock-dependent rhythm activation of signal pathways related to its regulation^22^. Therefore, we speculate that the ribosome-related pathways or terms enriched by genes with high expression levels in the sleep state are likely to be intuitively reflected by the regulation of the circadian clock in the brain, and the ribosome synthesis-related genes present a state of high expression level in the sleep state.

### Changes of important physiological processes in bat brain before and after feeding

To better explain the results, we used the “wake state” as a “before feeding state”, and the “rest state” as an “after feeding state”. The results of GO and KEGG analyses together indicated that the physiological processes related to drug metabolism are active after feeding for *V. sinensis*, and they were significantly enriched in 63 highly expressed genes. This pattern of changes in the expression of genes in the brain of *V. sinensis* is likely to reflect the adaptive evolution of bats over a long period of time due to their special diets. The food composition of bats is complex, and insects are the food component for most bat species^23^. According to the analyses of feces, the food composition of *V. sinensis* is mainly Coleoptera and Lepidoptera insects, and these insects are often concentrated in large numbers in areas of cultivated land^12^. Insects may have eaten plants containing pesticides, and owing to bioaccumulation, the pesticides can be accumulated in the insects and then in the predatory *V. sinensis*. Therefore, the physiological activity of drug metabolism in bats after feeding being higher than before eating is likely to be a self-protection mechanism for their bodies. In addition, our research also found that after bats feed, the pathways related to immune regulation in the brain are also enriched by genes with high expression levels and present an active state. We speculate that after bats feed, they also need to mobilize the immune regulation processes in the body to combat body damage caused by food ingredients and food contamination.

In comparison of bats before and after feeding, various endocrine activity-related pathways were significantly enriched by high expression levels of genes and showed a high level of endocrine activity, for example, the renin-angiotensin system, renin secretion, and cortisol synthesis and secretion. Renin is a component of the renin-angiotensin system and can produce angiotensin II (AngII)^24^. In the central nervous system, AngII can regulate insulin-stimulating and sodium appetite responses^25^. The renin-angiotensin system (RAS) plays an important role in the regulation of blood pressure, and it has a significant circadian rhythm^19^. Before feeding, *V. sinensis* may actively adjust the blood pressure and blood sugar through various endocrine-related physiological processes to prepare for predation activities. This may also be the result of adaptive evolution in bats.

### Daily rhythms of gene expression and physiological patterns in the bat brain

Accordingly, six rhythmic expression genes were detected in the brain of bat, which may indicate potential activity changes of related physiological processes. In the trend analysis module 6, 27 genes could be significantly clustered and showed similar expression patterns. The expression levels of these genes began to decrease in the after feeding phase (4:00), were the lowest in the sleep phase (10:00), and gradually increased in the before feeding phase (16:00) to the activity phase (22:00), where the expression levels were highest. Combined with previous studies, it is not difficult to imagine that the changes in daily expression levels of these genes in the brain are similar to the daily rhythms of other nocturnal rodents, but opposite to the daily rhythms of diurnal mammals. Therefore, our hypothesis is that the trend changes of the genes in module 6 at the four time points are consistent with the biological habits of *V. sinensis* that hide by day and come out at night. In addition, these genes were significantly enriched in cortisol synthesis and secretion pathways, aldosterone synthesis and secretion, and circadian rhythm pathways. Therefore, we can infer from these pathways that the physiological processes related to endocrine and circadian rhythms regulation in the brain of *V. sinensis* have clear daily periodic changes that regulate the overall behavioral rhythms of bats.

We also found that *Per1* may be an important clock gene in the brain of *V. sinensis*, and the gene has obvious characteristics of daily rhythmic expression. The *Per1* gene was differentially expressed in the sleep vs activity comparison, and the trend analysis results showed that the gene also has significant daily rhythmic expression characteristics. More importantly, the results of GO suggested that *Per1* was significantly enriched with various terms related to circadian rhythms, including regulation of circadian rhythm, circadian regulation of gene expression, and entrainment of circadian clock by photoperiod, and the circadian rhythm and circadian rhythm-fly pathways were significantly enriched by *Per1* in the KEGG results. Previous research demonstrated that *Per* is an important core circadian clock gene, and it was the earliest circadian clock gene to be cloned in *Drosophila*^26^. The mutation of *Per* can cause changes in the rhythm of *Drosophila*, so the gene was named "Period"^26^; the gene is involved in the regulation of the periodic expression of related genes in the brain. In mammals, three *Per* proteins have been identified: *Per1*, *Per2* and *Per3*, the *Per1* can participate in the input channel of circadian clock and play an important role in the daily rhythm of light signal input^27^. Studies have shown that *Perl* and *Per2* mutant mice lost free oscillations in total darkness^28^. In addition, *Per1* is orthologous to a key clock gene in other animals, so we are expected to be important for circadian clock in *V. sinensis*. This study is the first to discover an important role of *Per1* in regulating the daily rhythms and physiological processes in the brains of bats, revealing that bats may have the same molecular basis of circadian clock regulation as other nocturnal animals.

As is well known, the molecular mechanism of the circadian clock is a complex network^2^; in addition to *Per*, there are also core circadian clock genes such as *Clock, Cry, Bmal1,* and *Reverb* that participate in circadian clock regulation of mammals. In the mole-rat, *Bmal1*and *Per2* peaked at the same time in the morning, and this phase shift is considered to be fundamental for circadian clock function^29^. There are some genes related to circadian rhythms and sleep in owls, such as *CRY1*, *CPT1A*, and *STAR. CRY1* is a central component of the circadian clock^30,31^. *STAR* plays a role in the regulation of steroid hormone synthesis by mediating the transport of cholesterol through the mitochondrial membrane, and *CPT1A* encodes a key protein for the mitochondrial oxidation of long-chain fatty acids and is linked to the GO term "circadian rhythm" and the “circadian clock” pathway in humans^31^. Although these core circadian clock genes were not significantly differentially expressed at the four time points, this does not mean that these genes are not involved in the regulation of bat circadian rhythms. This needs to be further examined through detailed experiments and analysis.

## Conclusion

In summary, various physiological processes in the bat brain area showed different degrees of rhythmic variations, including various endocrine hormones, signal pathways, and transport activities. This study found that *Per1* may be an important circadian clock gene, exhibiting rhythmic expression patterns and may also involve in the regulation of the daily rhythms in the brain of *V. sinensis*. In consideration of the wild living individuals used in this study, we can only identify daily rhythms in genes expression, however, not the circadian rhythms related with circadian clock genes. A more detailed functional analyses and adaptive evolutionary analyses of circadian clock genes, like *Per1*, could be conducted in the future, to detect their important roles in the special daily rhythms of bats. Although only four time points in the bat were selected for analysis in this study, the results reflected the overall genetic basis of the daily rhythms in the brain of *V. sinensis*. In future research, we may consider adding more time points for sampling within a day and a multi-day setting to systematically reveal the molecular basis of bat daily rhythms.

## Materials and methods

### Ethics statement

According to the regulations of Wildlife Conservation of the People’s Republic of China (Chairman Decree (2016) No. 47), permits are required only for species included in the list of state-protected and region-protected wildlife species. Asian particolored bats used in this study are widespread in China and are not an endangered or region-protected animal species, and thus no specific permission was required. All the studies have been approved by Laboratory Animal Welfare and Ethics Committee of Jilin Agricultural University. All efforts were made to minimize suffering of animals. We confirm that we have complied with the ARRIVE guidelines, and all experiments were performed in accordance with relevant named guidelines and regulations.

### Sample collection

All animals used in this study were caught during July 2020 in Heilongjiang Province, China (45°32′55″N, 127°32′59″E). Throughout a week of observation, we recorded and confirmed the daily activities of Asian particolored bats. From approximately 2 a.m. to 6 a.m., bats successively returned back to their habitats. Bats could groom or emit calls sporadically; and we defined rest for this period of time. From 8 a.m. to 12 a.m., bats fall asleep, and we defined sleep for this period of time. From approximately from 2 p.m. to 6 p.m., most bat individuals wake up and perform simple activities such as emitting calls and fluttering; this period of time was defined as wakefulness. From approximately from 8 p.m. to 12 p.m., most bats fly out for hunting, and this period was defined as active. Therefore, four representative sampling time points were set corresponding to the four typical behaviors of bats in a day. These time points were 4 a.m., 10 a.m., 4 p.m., and 10 p.m., corresponding to the rest state (after feeding), sleep state, wake state (before feeding), and active state of the bat, respectively. Note that the wake state also represents the state before feeding, and the rest state represents the state after feeding.

Three biological repeats were included for each time point, and 12 individuals were collected in total. All sampling procedures at the four time points were completed within one day. For the states of rest, sleep, and wake, bats were captured by hand-draft nets, and for the active state, bats were captured by mist nets. To avoid any influence of sex-related differences, only females were selected for inclusion in the study. Animals were sacrificed by decapitation at the four time points of a day during the course of the study to avoid unnecessary suffering. The brain tissues from each individual were collected and immediately flash-frozen in liquid nitrogen followed by placement in a − 80 °C freezer until processing for total RNA isolation.

### RNA extraction

Total RNA was isolated using the Total RNA Extractor reagent in accordance with the manufacturer’s protocol. The quantity and quality of total RNA were measured using a Qubit 2.0 Fluorometer and gel electrophoresis. RNA samples of the same volume and concentration were used during the step of converting mRNA into cDNA. Three paired-end cDNA libraries of each time point were generated using the mRNA-Seq assay. In total, 12 cDNA libraries were prepared at an equimolar ratio for transcriptome sequencing on the Illumina HiSeq 4000 platform. All raw reads were deposited in the NCBI Short Read Archive (SRA) Database under SRA accession PRJNA756631.

### Transcriptome assembly and functional annotation

The raw reads were filtered by Trimmomatic software^32^ using four criteria: removing reads with adaptors; removing reads with unknown “N” bases; removing low-quality reads containing more than 50% low-quality bases (Q-value ≤ 20), and removing reads whose length was less than 35 nt. To construct a general reference transcriptome for subsequent analysis, the cDNA library sequences of 12 liver tissues sampled at the same time point and the brain tissue sequences were put together for splicing and assembly. The specific liver data analysis is published elsewhere. All high-quality raw reads from 12 individual cDNA libraries were used for de novo assembly by Trinity software^33^ with the default parameters. After the transcripts were reduced for sequence redundancy, the longest transcript in each cluster was taken as a unigene for further analysis. The assembly result was evaluated by parameters such as the longest value of the sequence, the shortest value of the sequence, the N50 value, and the N90 value.

Unigene annotations included protein function annotations, KOG function annotations, and Gene Ontology (GO) and pathway annotations. In detail, we used Blast + ^34^ on the NCBI nonredundant protein (Nr) database, the Swiss-Prot protein database, the KOG database and the Kyoto Encyclopedia of Genes and Genomes (KEGG) database. The GO annotation of unigenes was based on the protein annotation results of Swissprot and TrEMBL and the annotation information of Uniprot.

### Differential expression analysis

Considering the differences in library size, we first performed inter-sample normalization and used Bowtie2 software^35^ to map clean reads to the assembled reference sequence. Then, we used Salmon software^36^ to locate the genomic region or gene exon regions and estimated the gene expression levels by counting the reads. For the sequencing depth and gene length as well as the impact of the sample on the read count, the TPM value (Transcripts Per Million) was applied to determine the level of expression of each gene^37^.

The correlation coefficient between each pair of replicates for the four time points was calculated using the R package (version 2.16.2) to evaluate the reliability of the experimental results as well as the operational stability. To determine the separation of expression patterns across samples, PCA was performed on the levels of all unigenes using R package (gmodels, version 3.4.1).

The differentially expressed genes (DEGs) at the four time points were evaluated by DESeq software^38^. To clarify the differences in the transcription levels of genes in different activity states of *V. sinensis*, we compared the transcriptome data of two adjacent time points and obtained four comparisons of DEGs. In addition, we also analyzed the rest vs wake and sleep vs activity comparisons, and a total of six pairwise comparisons were obtained. Among these, we were interested in the comparisons of rest vs wake and sleep vs activity. The rest vs wake comparison represents the physiological state changes between after feeding vs before feeding, which may reflect the changes of rhythms gene expression caused by feeding behavior, and the sleep vs activity comparison represents the changes between the daytime and nighttime brain transcriptomes. In terms of the definitions of upregulation and downregulation of specific genes in each pairwise comparison, an upregulated gene was defined as one that had a higher expression level in the former time point than in the latter and vice versa for a down-regulated gene.

To create a list of high-confidence DEGs for further analyses, the following stringent criteria were used: fold change > 2 and adjusted *p* value < 0.05^39^. The visualization of the DEGs in the brain tissue collected at four time points was presented by creating heatmaps with the GPLOTS R package.

### GO category and KEGG pathway enrichment analyses

Downstream functional classification was achieved through the integrated localization of GO^40^ and KEGG pathway databases^41–43^. We conducted the GO and KEGG enrichment analysis on the DEGs in OmicShare (http://www.omicshare.com/). All *p* values were computed using the hypergeometric test, and multiple test correction was performed using the Benjamini–Hochberg method based on an FDR (false discovery rate) cut-off of 0.05.

### Downstream rhythmic analysis of DEGs

In order to determine the gene expression patterns at the four time points over a day, trend analysis was conducted using the STEM—Short Time-series Expression Miner software^44^ in OmicShare (http://www.omicsshare.com/). We constructed a gene set combining the six groups of DEGs, and 557 genes were obtained after removing duplicates which were detected in more than two pairwise comparisons. Those 557 genes were all differentially expressed genes with their expressed values at the four sampling time points. To determine the rhythmic changes of the important physiological and biochemical processes involved in the genes at four time points, we performed GO and KEGG enrichment analyses on the genes in the significant trend module.

Furthermore, for those 557 genes, we have performed JTK_CYCLE and ARSER analyses using the metacycle package by the R software to identify rhythmic genes, adjusted *p* value < 0.05 was used as the stringent criteria^45^.

### Identification of key circadian rhythm-related terms, pathways, and genes

We focused on the circadian rhythm-related terms and pathways among those that were significantly enriched by genes from the trend analysis and six comparative comparisons. Considering the important functions in the regulation of circadian rhythm, the genes enriched in circadian-related terms and pathways were assumed to be the most important daily rhythm clock genes in bat brains. To determine the daily rhythm clock genes among the enriched genes, we searched for detailed information in published research articles based on their basic annotation information to identify their potential functions in the bat brain.

### Validation of sequencing data by qPCR.

We randomly selected 16 genes from the 557 differentially expressed genes to test the validity of the expression patterns detected by RNA-Seq analysis. *Per1* was included and β-actin and 18S are selected as the house-keeping genes. The information of those genes, including the sequences of their primer pairs are listed in Supplementary Table S6. Three replicates were analyzed per time point using qPCR on the Applied Biosystems StepOne Real-Time PCR System (Applied Biosystems). Complementary DNAs (cDNAs) were synthesized using the same RNA samples as used for RNA-Seq, and 1 µg of total RNA was reverse-transcribed using reverse transcriptase. The TransStart Top Green qPCR SuperMix (Trans) was used for qPCR reactions. The final reaction volume of 20 μl included 1 μl of cDNA, 10 μl of 2 × TransStart Top Green qPCR Supermix, 0.4 μl of each primer, 0.4 µl of 50 × ROX reference dye and 7.8 μl of ddH_2_O. The PCR was performed under the following conditions: pre-denaturation at 94 °C for 30 s; then 40 cycles of 94 °C for 5 s, 60 °C for 30 s with data collection after each cycle, followed by the application of a melting curve. The amplification efficiencies of the house-keeping genes and 16 target genes were all between 90 and 100%. The relative expression levels of each target gene were calculated against two house-keeping genes, β-actin and 18S, by using the 2^−ΔΔCT^ method^46^. Regression analysis was performed to compare the expression values from the qPCR with the RNA-Seq results.

## Supplementary Information

Supplementary Information 1.

Supplementary Information 2.

Supplementary Information 3.

Supplementary Information 4.

Supplementary Information 5.

Supplementary Information 6.

Supplementary Information 7.

## Data Availability

All data generated or analyzed during this study are included in this paper (and its Supplementary Information files S1).

## References

[1] Su Y, Foppen E, Mansur Machado FS, Fliers E, Kalsbeek A (2018). The role of the daily feeding rhythm in the regulation of the day/night rhythm in triglyceride secretion in rats. Chronobiol. Int..

[2] Pilorz V, Astiz M, Heinen KO, Rawashdeh O, Oster H (2020). The concept of coupling in the mammalian circadian clock network. J. Mol. Biol..

[3] Refinetti R (1999). Relationship between the daily rhythms of locomotor activity and body temperature in eight mammalian species. Am. J. Physiol..

[4] Xia C, Zhang Y (2019). Lack of daily heart rate rhythms in Adelie penguin chicks during the polar day. Polar Rec..

[5] Christie AE (2018). Circadian signaling in Homarus americanus: Region-specific de novo assembled transcriptomes show that both the brain and eyestalk ganglia possess the molecular components of a putative clock system. Mar. Genomics.

[6] von Schantz M, Lucas RJ, Foster RG (1999). Circadian oscillation of photopigment transcript levels in the mouse retina. Brain Res. Mol. Brain Res..

[7] Mure LS (2018). Diurnal transcriptome atlas of a primate across major neural and peripheral tissues. Science.

[8] Verwey M, Amir S (2009). Food-entrainable circadian oscillators in the brain. Eur. J. Neurosci..

[9] Challet E, Mendoza J (2010). Metabolic and reward feeding synchronises the rhythmic brain. Cell Tissue Res..

[10] Arita HT, Fenton MB (1997). Flight and echlocation in the ecology and evolution of bats. Trends Ecol. Evol..

[11] Peixoto FP, Braga PHP, Mendes P (2018). A synthesis of ecological and evolutionary determinants of bat diversity across spatial scales. Bmc Ecol..

[12] Yin Z (2020). Changes in the gut microbiota during Asian particolored bat (Vespertilio sinensis) development. PeerJ.

[13] Goldsmith, C. S. & Bell-Pedersen, D. in *Advances in Genetics, Vol 84* Vol. 84 *Advances in Genetics* (eds T. Friedmann, J. C. Dunlap, & S. F. Goodwin) 1–39 (2013).

[14] Ko ML, Jian K, Shi L, Ko GYP (2009). Phosphatidylinositol 3 kinase-Akt signaling serves as a circadian output in the retina. J. Neurochem..

[15] Born J, Wagner U (2004). Memory consolidation during sleep: role of cortisol feedback. Ann. N. Y. Acad. Sci..

[16] Owen MA, Czekala NM, Swaisgood RR, Steinman K, Lindburg DG (2005). Seasonal and diurnal dynamics of glucocorticoids and behavior in giant pandas. Ursus.

[17] Lightman SL, Birnie MT, Conway-Campbell BL (2020). Dynamics of ACTH and cortisol secretion and implications for disease. Endocr. Rev..

[18] Thosar SS (2019). Separate and interacting effects of the endogenous circadian system and behaviors on plasma aldosterone in humans. Am. J. Physiol. Regul. Integr. Compar. Physiol..

[19] Hilfenhaus M (1976). Circadian rhythm of the renin-angiotensin-aldosterone system in the rat. Arch. Toxicol..

[20] Fleming PA, Gray DA, Nicolson SW (2004). Circadian rhythm of water balance and aldosterone excretion in the whitebellied sunbird Nectarinia talatala. J. Compar Physiol. B Biochem. Syst. Environ. Physiol..

[21] Jouffe C (2013). The Circadian Clock Coordinates Ribosome Biogenesis. Plos Biol..

[22] Missra A (2015). The circadian clock modulates global daily cycles of mRNA ribosome loading. Plant Cell.

[23] Cohen Y, Bar-David S, Nielsen M, Bohmann K, Korine C (2020). An appetite for pests: Synanthropic insectivorous bats exploit cotton pest irruptions and consume various deleterious arthropods. Mol. Ecol..

[24] Share L (1979). Interrelations between vasopressin and the renin-angiotensin system. Fed. Proc..

[25] Fitzsimons JT (1984). The renin-angiotensin system and sodium appetite. J. Physiol..

[26] Price, J. L. in *Circadian Rhythms* Vol. 393 *Methods in Enzymology* (ed M. W. Young) 35–60 (2005). 10.1016/S0076-6879(05)93048-6 15817283

[27] Benstaali C, Mailloux A, Bogdan A, Auzeby A, Touitou Y (2001). Circadian rhythms of body temperature and motor activity in rodents their relationships with the light-dark cycle. Life Sci..

[28] Verwey M, Khoja Z, Stewart J, Amir S (2008). Region-specific modulation of PER2 expression in the limbic forebrain and hypothalamus by nighttime restricted feeding in rats. Neurosci. Lett..

[29] Schoettner K, Oosthuizen MK, Broekman M, Bennett NC (2006). Circadian rhythms of locomotor activity in the Lesotho mole-rat, Cryptomys hottentotus subspecies from Sani Pass South Africa. Physiol. Behav..

[30] Cho YS (2019). Raptor genomes reveal evolutionary signatures of predatory and nocturnal lifestyles. Genome Biol..

[31] Borges R (2015). Gene loss, adaptive evolution and the co-evolution of plumage coloration genes with opsins in birds. BMC Genomics.

[32] Bolger AM, Lohse M, Usadel B (2014). Trimmomatic: a flexible trimmer for Illumina sequence data. Bioinformatics.

[33] Haas BJ (2013). De novo transcript sequence reconstruction from RNA-seq using the Trinity platform for reference generation and analysis. Nat. Protoc..

[34] Altschul SF (1997). Gapped BLAST and PSI-BLAST: a new generation of protein database search programs. Nucleic Acids Res..

[35] Langmead B, Salzberg SL (2012). Fast gapped-read alignment with Bowtie 2. Nat. Methods.

[36] Patro R, Duggal G, Love MI, Irizarry RA, Kingsford C (2017). Salmon provides fast and bias-aware quantification of transcript expression. Nat. Methods.

[37] Alvarez RV, Pongor LS, Marino-Ramirez L, Landsman D (2019). TPMCalculator: One-step software to quantify mRNA abundance of genomic features. Bioinformatics.

[38] Wang L, Feng Z, Wang X, Wang X, Zhang X (2010). DEGseq: An R package for identifying differentially expressed genes from RNA-seq data. Bioinformatics.

[39] Korthauer K (2019). A practical guide to methods controlling false discoveries in computational biology. Genome Biol..

[40] Harris MA (2004). The Gene Ontology (GO) database and informatics resource. Nucleic Acids Res..

[41] Kanehisa M, Goto S (2000). KEGG: kyoto encyclopedia of genes and genomes. Nucleic Acids Res..

[42] Kanehisa M (2019). Toward understanding the origin and evolution of cellular organisms. Protein Sci..

[43] Kanehisa M, Furumichi M, Sato Y, Ishiguro-Watanabe M, Tanabe M (2021). KEGG: Integrating viruses and cellular organisms. Nucleic Acids Res..

[44] Ernst J, Bar-Joseph Z (2006). STEM: a tool for the analysis of short time series gene expression data. BMC Bioinformatics.

[45] Wu G, Anafi RC, Hughes ME, Kornacker K, Hogenesch JB (2016). MetaCycle: an integrated R package to evaluate periodicity in large scale data. Bioinformatics.

[46] Livak, K. J. & Schmittgen, T. D. Analysis of relative gene expression data using real-time quantitative PCR and the 2(-Delta Delta C(T)) Method. *Methods (San Diego, Calif.)* **25**, 402–408 (2001). 10.1006/meth.2001.1262 11846609

