# Supplementary material for: Comparative analysis of the daily brain transcriptomes of Asian particolored bat
Source: Sci Rep. 2022 Mar 9;12:3876. doi: 10.1038/s41598-022-07787-z (PMC8907190; doi:10.1038/s41598-022-07787-z)
Supplement: Supplementary file 7 — Supplementary Information 7. [file 41598_2022_7787_MOESM7_ESM.pdf]

Comparative analysis of the daily brain transcriptomes of Asian particolored bat

Guoting Zhang, Yujia Chu, Tinglei Jiang, Jingjing Li, Lei Feng, Hui Wu, Hui Wang & Jiang Feng

Table S1. GO cell component enrichment data of six pairwise comparisons.
Table S2. GO molecular function enrichment data of six pairwise comparisons.
Table S3. GO biological process enrichment data of six pairwise comparisons.
Table S4. KEGG enrichment data of six pairwise comparisons.
Table S5. GO and KEGG enrichment data of module 6 (27 genes).
Table S6. Summary of qPCR primer sequences for Asian particolored bat
Figure S1. Venn diagram of Nr, Swiss-Prot, KOG, and KEGG databases showing homology sequence numbers of the unigenes in the four different databases.
Figure S2. KOG function classification of the unigenes. A total of 25 categories were obtained. The x-axis indicates the categories, and the y-axis indicates the numbers of the unigenes.
Figure S3. Correlation analysis between each pair of replicates at the four time points.

Figure S4. Principal component analysis (PCA) of the transcriptome of four time points.

Figure S5. The trend analysis module ordered based on p value significance of number of genes assigned versus expected. The gray trend module indicates that genes are significantly clustered into this module.
Figure S6. The circadian rhythm pathway [41]. The pathway ID is ko04710. The red frame in the figure shows the position of PER in the pathway.

Figure S1.

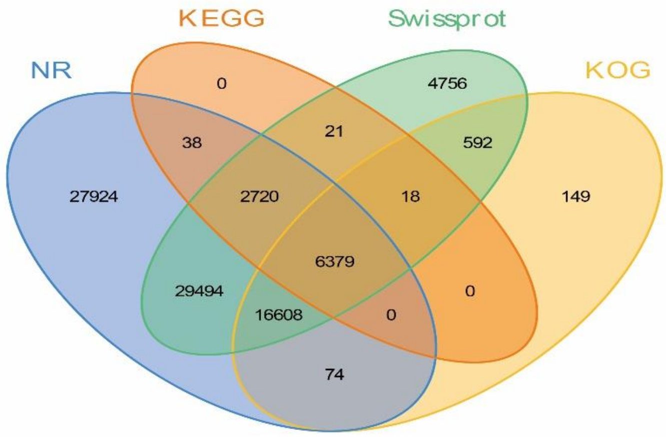

NR
KEGG
Swissprot
KOG
0
4756
38
21
592
27924
2720
18
149
6379
29494
0
16608
0
74

Figure S2.

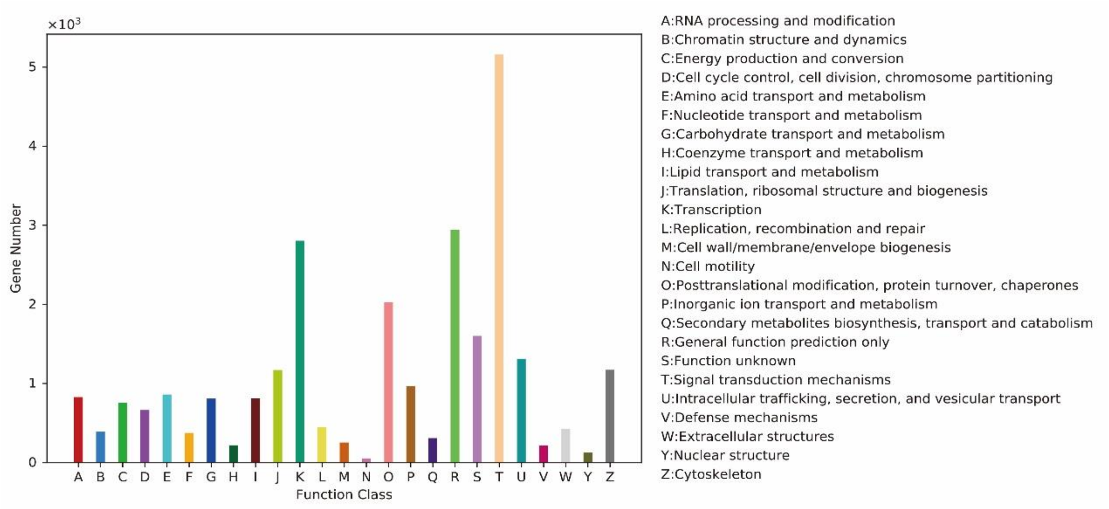
×10³
Gene Number
0
1
2
3
4
5
A B C D E F G H I J K L M N O P Q R S T U V W Y Z
Function Class
A:RNA processing and modification
B:Chromatin structure and dynamics
C:Energy production and conversion
D:Cell cycle control, cell division, chromosome partitioning
E:Amino acid transport and metabolism
F:Nucleotide transport and metabolism
G:Carbohydrate transport and metabolism
H:Coenzyme transport and metabolism
I:Lipid transport and metabolism
J:Translation, ribosomal structure and biogenesis
K:Transcription
L:Replication, recombination and repair
M:Cell wall/membrane/envelope biogenesis
N:Cell motility
O:Posttranslational modification, protein turnover, chaperones
P:Inorganic ion transport and metabolism
Q:Secondary metabolites biosynthesis, transport and catabolism
R:General function prediction only
S:Function unknown
T:Signal transduction mechanisms
U:Intracellular trafficking, secretion, and vesicular transport
V:Defense mechanisms
W:Extracellular structures
Y:Nuclear structure
Z:Cytoskeleton

Figure S3.

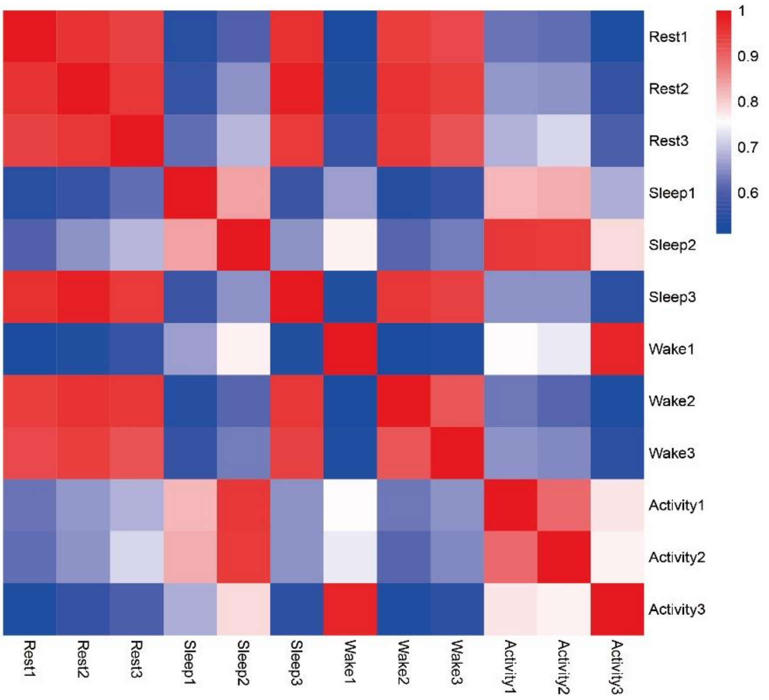
1
0.9
0.8
0.7
0.6
Rest1
Rest2
Rest3
Sleep1
Sleep2
Sleep3
Wake1
Wake2
Wake3
Activity1
Activity2
Activity3

Figure S4.

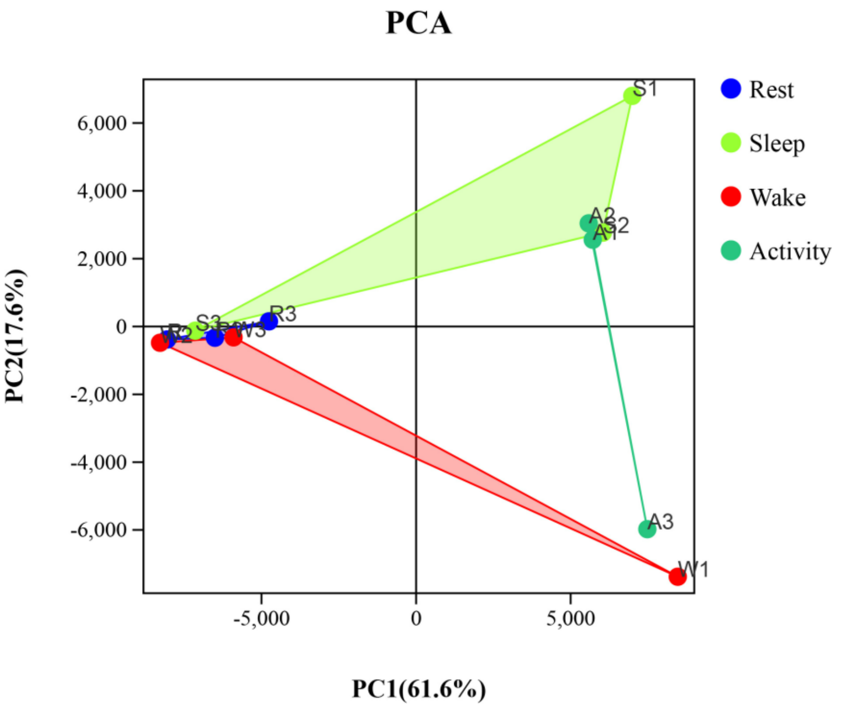

PCA
Rest
Sleep
Wake
Activity
PC2(17.6%)
PC1(61.6%)
6,000
4,000
2,000
0
-2,000
-4,000
-6,000
-5,000
0
5,000
S1
A2
S2
A1
R3
S3
W3
A3
W1

Figure S5.

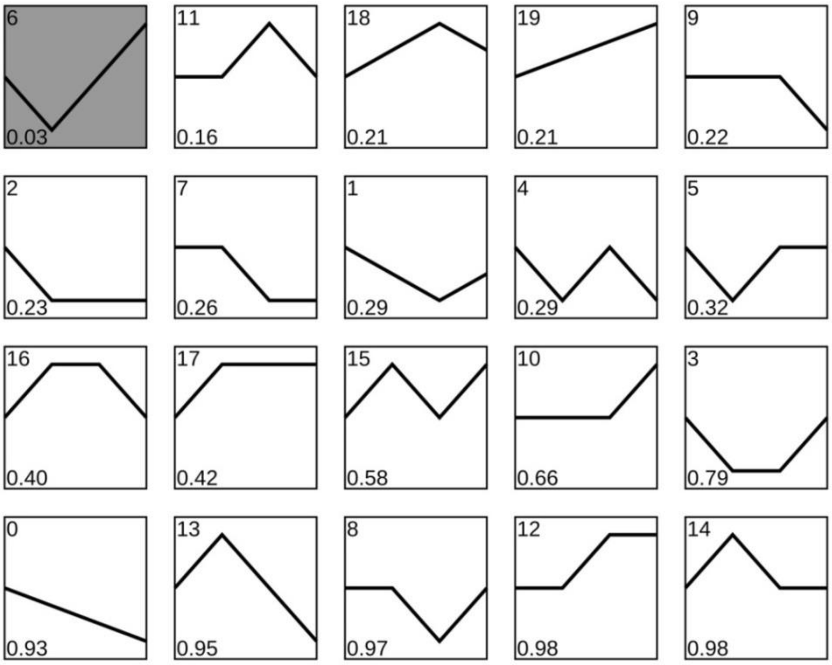
6
0.03
11
0.16
18
0.21
19
0.21
9
0.22
2
0.23
7
0.26
1
0.29
4
0.29
5
0.32
16
0.40
17
0.42
15
0.58
10
0.66
3
0.79
0
0.93
13
0.95
8
0.97
12
0.98
14
0.98

Figure S6.

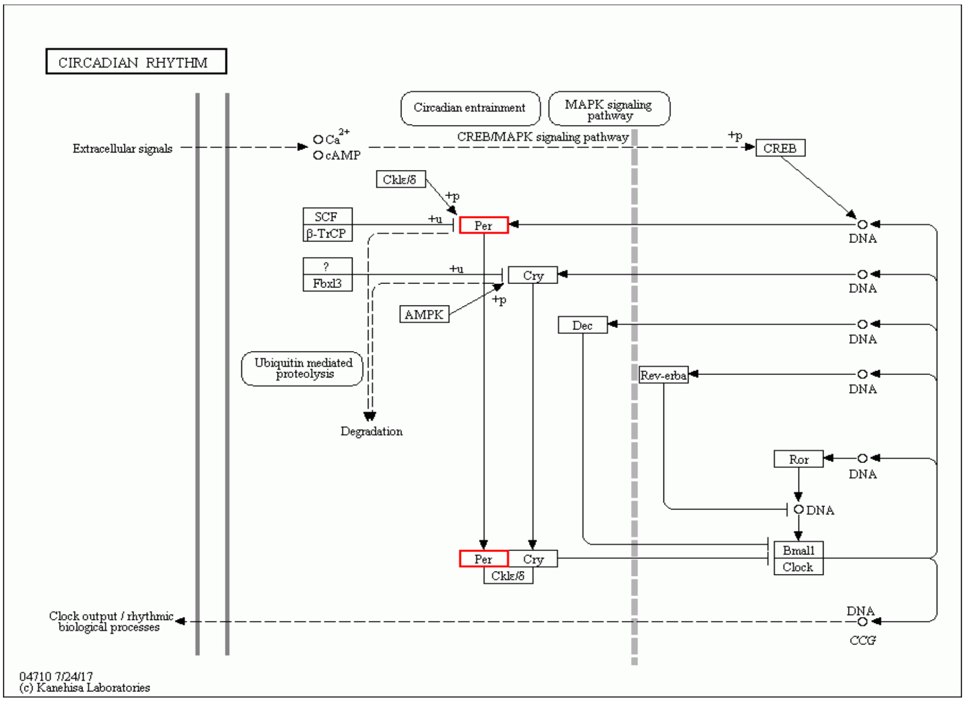

CIRCADIAN RHYTHM
Circadian entrainment
MAPK signaling pathway
Extracellular signals
Ca2+
cAMP
CREB/MAPK signaling pathway
+p
CREB
CkIε/δ
+p
SCF
β-TrCP
+u
Per
DNA
?
Fbxl3
+u
Cry
DNA
AMPK
+p
Dec
DNA
Ubiquitin mediated proteolysis
Rev-erba
DNA
Degradation
Ror
DNA
DNA
Bmal1
Clock
Per
Cry
CkIε/δ
Clock output / rhythmic biological processes
DNA
CCG
04710 7/24/17
(c) Kanehisa Laboratories
